# Simplified production and concentration of lentiviral vectors to achieve high transduction in primary human T cells

**DOI:** 10.1186/1472-6750-13-98

**Published:** 2013-11-12

**Authors:** Adam P Cribbs, Alan Kennedy, Bernard Gregory, Fionula M Brennan

**Affiliations:** 1 Kennedy Institute of Rheumatology, Roosevelt Drive, Headington OX3 7FY, Oxford, UK

**Keywords:** Titre, Primary lymphocytes, CD45RA^+^, Ultracentrifugation

## Abstract

**Background:**

Lentiviral vectors have emerged as efficient vehicles for transgene delivery in both dividing and non-dividing cells. A number of different modifications in vector design have increased biosafety and transgene expression. However, despite these advances, the transduction of primary human T cells is still challenging and methods to achieve efficient gene transfer are often expensive and time-consuming.

**Results:**

Here we present a simple optimised protocol for the generation and transduction of lentivirus in primary human CD45RA^+^ T cells. We show that generation of high-titre lentivirus with improved primary T cell transduction is dependent upon optimised ultracentrifuge speed during viral concentration. Moreover, we demonstrate that transduction efficiency can be increased with simple modifications to the culturing conditions. Overall, a transduction efficiency of up to 89% in primary human CD45RA^+^ cells is achievable when these modifications are used in conjunction.

**Conclusion:**

The optimised protocol described here is easy to implement and should facilitate the production of high-titre lentivirus with superior transduction efficiency in primary human T cells without the need for further purification methods.

## Background

Replication-deficient pseudotyped lentiviral vectors are widely used as basic tools in research and have the potential to be utilised as therapeutic agents in severe immunodeficiencies [1,2]. Production of HIV-derived lentiviral vectors is performed by transfecting vectors harbouring self-inactivating long terminal repeat (LTR) regions together with the transgene on one vector (the transfer vector), while the additional transcripts required for packaging (Gag/Pro/Pol) and encapsulation (Env) are encoded on separate vectors [3]. In second-generation packaging systems, all packaging genes are encoded by one vector that is transfected along with the transfer vector and encapsulation vector to generate virions. In the third-generation packaging system the Rev gene is encoded on a separate packaging plasmid, leading to increased biosafety [4].

Concentration of lentiviral stocks is performed to increase lentivirus titre and remove impurities for superior transduction of target cells. Typically ultracentrifugation is used, although other techniques such as ultrafiltration [5-7] and ultracentrifugation over a density gradient [7,8] are also commonly used. Numerous studies have developed optimised lentiviral production protocols based on these procedures [6,9,10], however most approaches have focused their attention on obtaining high lentivirus titre, with little regard given to the infectivity of virus produced.

Efficient lentiviral transduction of numerous cell lines has been demonstrated previously [11]. In contrast, primary human T cells are refractory to transduction by lentivirus, with quiescent T cells being particularly problematic to transduce [12,13]. Furthermore, the completion of reverse transcription and integration into the genome does not occur unless T cells are activated through the TCR and/or by proliferative cytokines [14].

In this study we have generated high-titre lentivirus with superior transduction efficiency in primary human T cells. Specifically, we demonstrate that time consuming and costly additional purification methods are dispensable when lentivirus is concentrated using lower centrifuge speeds. This resulted in both increased lentiviral titres and increased infectivity in human primary T cells. Moreover, we show that transduction efficiency can be improved further when optimal conditions are used to culture primary T cells.

## Methods

### Cell lines

HEK 293T/17 cells (ATCC No. CRL-11268) were cultured in high glucose-containing Dulbecco’s modified Eagle’s medium (DMEM; Lonza) supplemented with 10% foetal calf serum (FCS, Gibco) at 37°C at 5% CO2, unless otherwise stated. Human T lymphocyte derived Jurkat cells (ATCC No. TIB-152) were cultured in RPMI 1640 (Lonza) supplemented with 5% FCS prior to transduction.

### Primary CD45RA T cell isolation and stimulation

Human peripheral blood mononuclear cells (PBMCs) were isolated from electrophoresis cones (North London Blood Transfusion Service) or healthy volunteers by Ficoll-Hypaque centrifugation. Human blood collections were taken following written informed consent and approval of the London Riverside research committee, REC number: 07/H0706/81 and in agreement with the Helsinki Declaration. Peripheral blood lymphocytes (PBLs) were isolated by centrifugal elutriation. CD4^+^CD45RA^+^ cells were isolated from PBLs by negative bead isolation (Miltenyi). Lymphocytes were stimulated with either plate bound anti-CD3 (OKT3 – Insight Biotechnology) and soluble anti-CD28 (BD Pharmingen) or anti-CD3/CD28 activation beads (Invitrogen). Cells were cultured in X-VIVO 15 (Lonza) and supplemented with either 5% FCS or 5% Human serum (Biosera), together with IL-2 (100 ng/mL) and IL-7 (15 ng/mL).

### Plasmid constructs

The pCCL-Empty plasmid was constructed from a previously characterised vector, MA1, in which two transgenes can be efficiently transcribed from a bi-directional origin: with the minimal cytomegalovirus promoter for ΔNGFR (a truncated form of the nerve growth factor receptor), and a second transgene transcribed from the human phosphoglycerate kinase promoter [15]. The pCCL-Empty plasmid was kindly provided by Prof M Levings [16]. The lentivirus packaging plasmids, psPAX2, pRSV-Rev, pMDLg/pRRE and the pMD2. G envelope plasmid containing VSV-G were kindly provided by D. Trono [4]. For lentivirus production, plasmids were prepared using the Endofree Plasmid Maxi kit (Qiagen) according to manufacturer’s protocol.

### Preparation of flasks for lentivirus production

Lentiviral vectors expressing the ΔNGFR gene were produced as follows. 24 hrs prior to transfection, 25 × 10^6^ 293T cells were plated onto poly-L-lysine (Sigma) coated 175 cm^2^ flasks. Two hrs prior to transfection, medium was replaced with RPMI 1640 supplemented with 5% FCS. All solutions were sterilised by filtration through 0.22 μm filters. Solutions were stored in aliquots at -20°C, and thawed prior to use.

### Standard lentivirus production and concentration procedure

The lentiviral transfer vector DNA, together with psPAX2 packaging and pMD2. G envelope plasmid DNA were combined at a ratio of 4:3:1, respectively. Production of 3^rd^ generation lentivirus was performed using the combined ratio of transfer plasmid, packaging plasmid, Env plasmid and pRSV-Rev plasmid at 4:2:1:1, respectively. The precipitate was formed by adding 80 μg of DNA to a final volume of 1.35 mL distilled H_2_O and 150 μl 2.5M CaCl_2_ (Sigma). A sterile 2 mL pipette was used to vigorously bubble air through the DNA mix, during which, 1.5 mL 2× HEPES-buffered saline (280 mM NaCl, 50 mM HEPES, 1.5 mM Na_2_HPO_4_, pH 7.0) was added dropwise into the precipitate. The solution was briefly vortexed and incubated at room temperature for 30 min. Following this, the solution was mixed again by gentle vortexing, and then added dropwise to the cells. Flasks were rocked gently in a circular motion to distribute the precipitates, and then returned to the incubator at 3% CO_2_ unless otherwise stated. Four to six hrs later, cells were gently washed once with PBS and fresh growth medium added. Sixteen hrs post-transfection, the medium was replaced with RPMI supplemented with 5% FCS unless otherwise stated and incubated at 5% CO_2_ for 24 hrs prior to the initial collection of viral supernatant. A second collection was made after a further 24 hrs. The conditioned medium from the two harvests was combined and cleared by centrifugation at 1500 rpm for 5 min at 4°C then passed through a 0.45 μm pore PVDF Millex-HV filter (Millipore).

Concentration of lentivirus using ultracentrifugation was performed with a Sorval Discovery 100 SE centrifuge using an AH-629 rotor. 30 mL of filtered lentivirus supernatant was added to 36 mL pollyallomer conical tubes (Beckman). Centrifugation was performed for 90 min at 90,000 g unless otherwise stated. Supernatant was completely removed and virus pellets resuspended in 300 μL PBS overnight at 4°C and stored at -80°C until use.

### Lentiviral transduction of primary T cells

Lentivirus was added to primary CD45RA^+^ lymphocytes cultured in X-VIVO media supplemented with 10% FCS (initial culturing conditions) or 10% HS (final optimal culturing conditions) and 6 μg/mL polybrene. Lentivirus was then added to give a multiplicity of infection (MOI) of 50. After overnight incubation, lentivirus was removed and fresh media added. Following 48 hrs (initial culturing conditions) or 24 hrs (final optimal culturing conditions) of culture the cells were removed and NGFR^+^ cells were determined by flow cytometry using antibody APC-CD271 (BD Pharmingen).

### Lipofectamine transfection

Where lipofectamine (Invitrogen) reagents were used for comparison of transfection methods, 80 μg plasmid DNA was again used in the same ratio as with the calcium phosphate method and transfections were carried out according to the manufacturer’s protocol.

### Acrodisc purification

Purification of lentivirus using a Mustang Q Acrodisc (PALL) was performed according to the manufacturer’s recommendations. Briefly, 50 mL aliquots of lentivirus supernatant were adjusted to 25 mM Tris-HCl, pH 8.9 and loaded onto a Mustang Q Acrodisc and filtered through the Acrodisc syringe filter. The Acrodisc was eluted using a step gradient ranging from 0.3 to 1.5 M NaCl in 25 mM Tris-HCl, pH 8.5. Filtered supernatant was then added to 36 mL pollyallomer conical tubes and ultracentrifuged at 20,000 g for 90 min.

### Optiprep purification

For concentration using Optiprep (Axis-shield), a 220 μL cushion of Optiprep was added to pollyallomer conical tubes and lentivirus-containing supernatant (30 mL) was gently layered into each tube taking care not to disturb the Optiprep. Samples were ultracentrifuged at 20,000 g for 90 min. Media above the media/Optiprep interface was carefully removed from each tube and discarded. The residual media containing lentivirus and Opitiprep was shaken at 200 rpm for 2 hrs in sealed tubes. The resulting mixture was pooled and centrifuged at 20,000 g for 24 hrs. Supernatant was discarded and the remaining pellet was resuspended in 300 μL of PBS, shaken overnight at 4°C, and then stored at -80°C until use.

### Titration

Titres were determined by transducing 1 × 10^5^ Jurkat cells in 100 μl of RPMI 1640 growth medium. Titres were determined in triplicate with serial dilutions from 1:10 to 1:1000 for un-concentrated vector stocks, or from 1:100 to 1:10000 for concentrated vector stocks in a final volume of 100 μl with 6 μg/mL polybrene (Sigma). The following day, plates were spun at 1,500 rpm for 5 min to remove cells from suspension and fresh growth medium added. After a further 48 hr incubation, cells were harvested and NGFR expression was determined by flow cytometry using APC-CD271 antibody (BD Pharmingen). Titres were determined based on the percentage of NGFR positive cells and calculated according to the following formula (F × N × D × 1,000)/V, where F = percentage cells positive for NGFR, N = number of cells at time of transduction (usually 1 × 10^5^ cells), D = fold dilution of vector used in transduction, V = volume (μl) of diluted vector sample in each well. The virus titre is expressed as transducing units/mL (TU/mL) ± standard error [5].

### Final simplified production and concentration of lentivirus

25 × 10^6^ 293T cells were plated onto poly-L-lysine (Sigma) coated 175 cm^2^ flasks. Two hrs prior to transfection, medium was replaced with RPMI 1640 supplemented with 5% FCS.

The lentiviral transfer vector DNA, together with packaging and envelope plasmid DNA were combined at a ratio of 4:1:3 respectively. The precipitate was formed by adding 80 μg of DNA to a final volume of 1.35 mL distilled H_2_O and 150 μl 2.5M CaCl_2_ (Sigma). A sterile 2 mL pipette was used to bubble air through the DNA mix, during which, 1.5 mL 2x HEPES-buffered saline (280 mM NaCl, 50 mM HEPES, 1.5 mM Na_2_HPO_4_, pH 7.0) was added dropwise into the precipitate. The solution was briefly vortexed and incubated at room temperature for 30 min. Following this, the solution was mixed again by gentle vortexing, and then added dropwise to the cells. Flasks were rocked gently in a circular motion to distribute the precipitates, and then returned to the incubator at 3% CO_2_ unless otherwise stated. Four to six hrs later, cells were gently washed once with PBS and fresh growth medium added. Sixteen hrs post-transfection, the medium was replaced with RPMI supplemented with 5% FCS unless otherwise stated and incubated at 5% CO_2_ for 24 hrs prior to the initial collection. Sodium butyrate (Sigma) was added 16 hr post-transfection into the culture medium at a final concentration of 1 mM. A second collection was made after a further 24 hrs. The conditioned medium from the two harvests was combined and cleared by centrifugation at 1500 rpm for 5 min at 4°C then passed through a 0.45 μm pore PVDF Millex-HV filter (Millipore).

Concentration of lentivirus using ultracentrifugation was performed with a Sorval Discovery 100 SE centrifuge using an AH-629 rotor. 30 mL of filtered virus supernatant was added to 36 mL pollyallomer conical tubes (Beckman). Centrifugation was performed for 90 min at 20,000 g. Supernatant was completely removed and virus pellets resuspended in 300 μL PBS overnight at 4°C and stored at -80°C until use.

### Statistical analysis

We used Graph Pad Prism 6 for analysis. We used one-way analysis of variance with Bonferroni correction and Student’s *t* test to determine differences between groups. The results presented are mean ± s.d. P < 0.05 was considered statistically significant.

## Results and discussion

### Comparison of lipofectamine and calcium phosphate transfection to generate lentivirus

**Figure 1:**
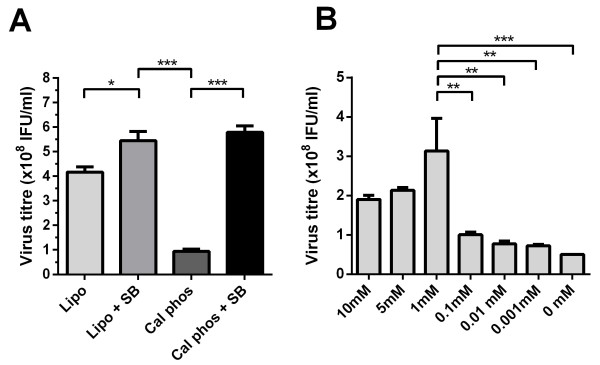
**High titre virus generated in 293T cells using calcium phosphate and sodium butyrate. A.** 293T cells were transfected with pCCL, psPAX2 and pMD2. G packaging plasmids using lipofectamine in the presence or absence of sodium butyrate, or calcium phosphate in the presence or absence of sodium butyrate. Data shown represents mean ± s.d. of six independent transfections **B.** Calcium phosphate transfection in 293T cells was performed using decreasing concentrations of sodium butyrate. Data represent mean ± s.d. of three independent experiments. *P < 0.05, **P < 0.01, ***P < 0.001 using one-way ANOVA with Bonferroni comparison.

In an initial set of experiments, the transfection efficiency of lipofectamine and a less expensive calcium phosphate based method, were compared. Calcium phosphate or lipofectamine transfection of HEK 293T cells was carried out in the presence or absence of 1 mM sodium butyrate, which has previously been shown to function as an enhancer of mammalian cell transfection efficiency [17]. As shown in Figure 1A, in the absence of sodium butyrate, the titres of lentivirus produced using lipofectamine-mediated transfection were significantly higher than those produced by the calcium phosphate based method. Addition of sodium butyrate resulted in a moderate improvement on virus titres produced by lipofectamine transfection. In contrast, a significant increase was observed in titres following sodium butyrate treatment of cells transfected by the calcium phosphate method. In fact, this resulted in titres comparable to those observed using the lipofectamine based method. Therefore all further transfections were carried out using the calcium phosphate and sodium butyrate transfection method.

In the previous experiments, use of sodium butyrate enhanced the titres of lentiviral vectors in all cases [18]. To determine the optimal concentration of sodium butyrate in the culture medium with the calcium phosphate transfection, lentiviral vector titres produced from cells cultured in medium containing a range of sodium butyrate concentrations were determined. As shown in Figure 1B, compared to non-supplemented transfections, the use of sodium butyrate increased virus titres when present at concentrations of 10 mM down to 1 μM in the culture medium. The highest viral titres were achieved using sodium butyrate at a concentration of 1 mM, with the presence of sodium butyrate at this concentration resulting in a ~6-fold increase in lentiviral titres compared to no sodium butyrate. Therefore for all further experiments 1 mM sodium butyrate was added at 16 hr post-transfection.

### Comparison of the second and third lentivirus packaging systems

**Figure 2:**
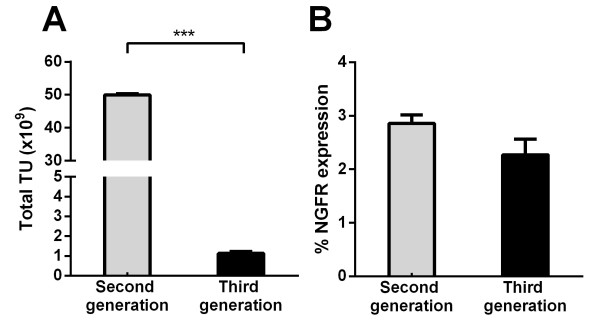
**Concentration of a second generation packaging system generates virus with superior transduction efficiency in primary CD45RA**^**+ **^**T cells. A.** The total transducing units generated using second and third generation packaging systems. **B.** The level of transduction for the second and third generation packaging systems in CD45RA^+^ T cells as measured by flow cytometry. Data represents mean ± s.d. of three independent preparations. ***P < 0.001 using Students t-test.

With the second-generation lentivirus packaging system, the Gag/Pro/Pol and Rev genes are encoded on a single plasmid and this was used, in addition to the transfer plasmid and envelope plasmid to produce lentivirus. In contrast, in the third-generation lentivirus packaging system, the Rev gene is encoded on a separate plasmid to the Gag/Pro/Pol genes, thereby necessitating the co-transfection of four plasmids as opposed to three for virus production [4]. As shown in Figure 2A, the second generation lentivirus vectors produced approximately fifty times more total yield of virus than the third generation. The ability to transduce difficult to infect cells such as primary T cells is dependent upon the ability to produce high viral yields with a sufficiently high titre [19]. Therefore we investigated the transduction efficiency of the second and third generation lentivirus systems in primary human CD45RA^+^ T cells. Following titration, equivalent MOIs were calculated for the second and third generation systems and used to transduce primary T cells. This revealed that the third-generation system did not have any beneficial effect on the ability of the viral vector to transduce these cells (Figure 2B). Therefore, due to the significantly higher viral yield obtained using the second-generation system, this system was used for the generation of all subsequent viral vector preparations.

### Comparison of lentivirus titre/transduction efficiency following ultracentrifugation and purification using Optiprep and Acrodisc

**Figure 3:**
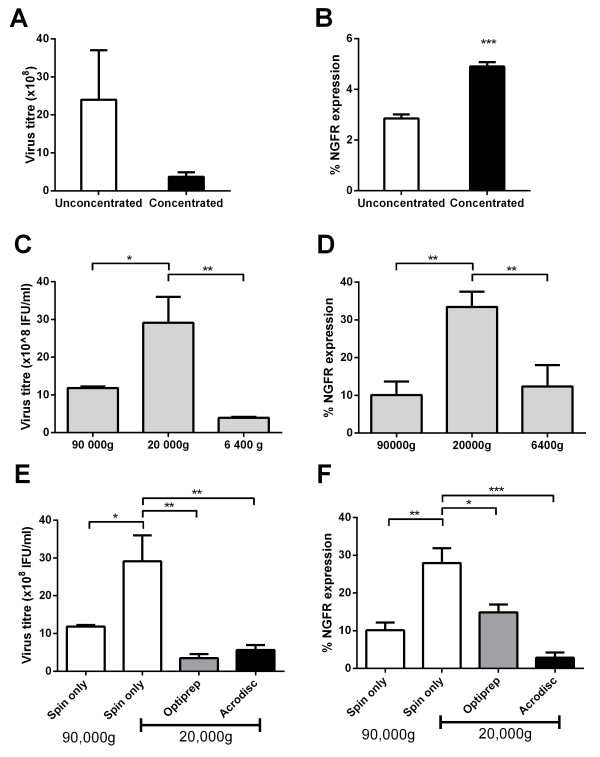
**Ultracentrifugation at 20 000 g improves virus titre and transduction efficiency in primary CD45RA**^**+ **^**T cells. A.** Virus titre measured before and after concentration by ultracentrifugation. **B.** The level of transduction using un-concentrated and concentrated lentivirus supernatant as measured by flow cytometry. **C.** Virus titre measured following centrifugation of viral supernatants at decreasing centrifugal speeds. **D.** Assessment by flow cytometry of transduction by lentivirus following concentration with decreasing centrifugal speeds. **E.** Virus titre measured following concentration of lentivirus using ultracentrifugation, Optiprep and Acrodisc methods. **F.** The level of transduction of each of the concentrated lentivirus stocks as measured by flow cytometry. *P > 0.05, **P > 0.01, ***P > 0.001 as determined by one-way ANOVA with Bonferroni correction or Students t-test. Data is represented as mean ± s.d. of three independent experiments and virus preparations.

It is commonplace to concentrate lentivirus by ultracentrifugation to obtain sufficient viral titres to transduce cells at a high MOI and remove contaminating impurities for sensitive *in vivo* applications. The standard procedure for concentrating lentivirus involves ultracentrifugation at 90,000 g for 90 min [20,21]. In order to use higher titres of lentivirus in our experiments we compared unconcentrated lentivirus with lentivirus concentrated by ultracentrifugation at 90,000 g for 90 min. We found that concentrating lentivirus resulted in a 10-fold reduction in virus yield, suggesting that significant amounts of virus are lost or lose infectivity during ultracentrifugation (Figure 3A). However, we showed a significant increase in the NGFR expression when primary CD45RA^+^ T cells are transduced at equivalent transducing units with the concentrated lentivirus (Figure 3B). This suggests that concentration removes impurities in the virus supernatant, which can enhance the transduction efficiency.

Concentrating lentivirus with reduced centrifuge speeds has previously been shown to increase the titre of lentivirus [22]; however whether this equates to increased transduction efficiency in primary T cells has never been demonstrated. Concentration of lentivirus is usually performed at 90,000 g [5,23], although some have shown that recovery of virus increases if the virus is centrifuged at a reduced speed of 20,000 g [9]. Similarly we showed a significant increase in virus titre (Figure 3C) and virus recovery (Additional file 1: Figure S1), which correlated with a significant increase in transduction in CD45RA^+^ T cells when virus was spun at 20,000 g (Figure 3D), suggesting that reduced spin speed improves virion stability and infectivity. Reduction of spin speed to 6,400 g resulted in a substantially reduced lentivirus titre and did not improve transduction of CD45RA^+^ T cells. These results suggest that optimisation of centrifugation speed is important to generate the highest titre of lentivirus with optimal infectivity and that high spin speeds may reduce virus recovery and lower transduction efficiency in primary human CD45RA^+^ T cells.

Having determined that concentrating virus at a lower speed results in improved lentivirus titres and transduction efficiency, we next determined whether we could improve lentivirus infectivity further by density gradient concentration (Optiprep) or ultrafiltration (Mustang Q Acrodisc). However, we found that using either Optiprep or Acrodisc concentration techniques resulted in a reduction in overall lentivirus titre (Figure 3E) and virus recovery (Additional file 1: Figure S2), which correlated with reduced transduction of primary CD45RA^+^ T cells (Figure 3F). These results suggest that centrifuging lentivirus at lower speeds negates the use of more costly and time-consuming purification techniques and is sufficient to remove impurities that may otherwise reduce transduction efficiency.

### Achieving high transduction of primary naïve T cells through modification of culturing conditions

**Figure 4:**
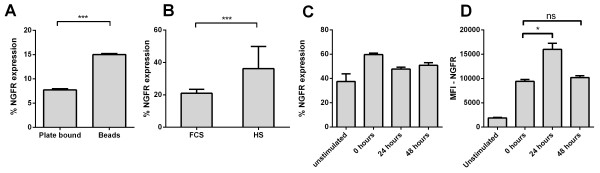
**Optimal culturing conditions for highly efficient transduction of primary CD45RA**^**+ **^**T cells. A.** Virus was generated using the standard production and concentration protocol. Primary CD45RA^+^ T cells were stimulated using plate bound anti-CD3 (5 μg/mL) and soluble anti-CD28 (1 μg/mL) or with CD3/CD28 activation beads (1 bead to 5 cells) then transduced following 24 hrs of stimulation. ***P < 0.001 as determined by Students t-test. Data represent mean ± s.d. of three independent experiments. **B.** Primary CD45RA^+^ T cells were stimulated with CD3/CD28 activation beads for 24 hrs then transduced and cultured in X-VIVO media supplemented with 10% FCS or 10% HS. ***P < 0.001 as determined by students t-test. Data represent mean ± s.d. of three independent experiments. **C** and **D.** Primary CD45RA^+^ T cells were stimulated using CD3/CD28 activation beads and cultured in X-VIVO media supplemented with 10% HS and transduced following 0, 24 and 48 hrs of activation. *P < 0.05 as determined by one-way ANOVA with Bonferroni correction. Data represent mean ± s.d. of three independent experiments.

Having optimised lentivirus production and concentration we next determined optimum culture conditions for transducing primary CD45RA^+^ T cells. For this optimisation we used lentivirus that had been produced using the ‘standard’ lentivirus production/concentration protocol as listed in the material and methods. Primary cells are notoriously difficult to transduce with lentivirus, although it has been shown that inducing cell-cycle entry into G_1b_ via stimulation of the T cell receptor allows increased transduction of naïve T cells [14,24]. This suggests that the strength of TCR signalling may be important for increased transduction efficiency. Therefore, we began to modify a standard protocol used to transduce primary T cells [16] (see material and methods for protocol). We determined whether stimulating CD45RA^+^ T cells with either plate bound anti-CD3 (5 μg/mL) and soluble anti-CD28 (1 μg/mL) or anti-CD3/CD28 activation beads (used at a 1:5 bead: cell ratio) led to increased transduction efficiency. We demonstrate that activating primary cells with anti-CD3/CD28 activation beads resulted in 1.94 fold increase in transduction efficiency (Figure 4A). Interestingly, this increase in transduction was associated with increased expression of activation markers CD25 and CD45RO^+^ (data not shown), suggesting that the cells were more activated. Our next step in optimisation was to determine whether the use of HS increased transduction efficiency. When human serum was used as a growth supplement, a significant increase in transduction was observed when compared to FCS cultured cells (Figure 4B), a modification that also led to increased activation marker expression (data not shown). One explanation for increased transduction efficiency could be that more cells are cycling, making them more amenable to transduction.

Having shown that transduction efficiency can be improved by using CD3/28 activation beads in combination with HS, we next determined transduction efficiency in unstimulated T cells and following 0, 24 and 48 hrs of TCR stimulation. There were no significant changes in the numbers of NGFR expressing cells between CD45RA^+^ cells that were transduced unstimulated or following stimulation with activation beads for 0, 24 or 48 hrs (Figure 4C). However a significant increase in the MFI of ΔNGFR was seen following transduction after 24 hrs of TCR stimulation (Figure 4D), suggesting that the cell cycle stage at which cells are transduced may be important for increased transgene expression, which is likely due to increased number of transgene integrations per cell.

### High transduction efficiency in primary T cells

**Figure 5:**
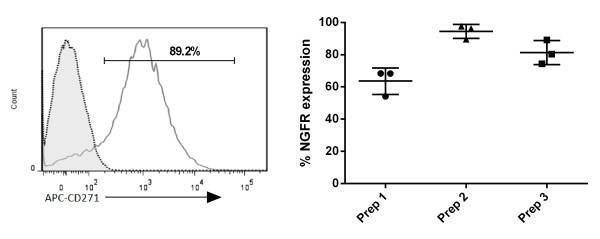
**High transduction of lentivirus in primary human T cells.** Primary CD45RA^+^ T cells were transduced using the ‘final’ simplified lentivirus generation and concentration protocol in combination with modifications made to the culturing conditions. Left panel shows representative histogram from one donor of NGFR expression following transduction with lentivirus. Right panel shows the percentage NGFR expression from three lentivirus preparations in nine independent experiments. Transduction efficiency was determined 24 hrs post-transduction using flow cytometry to determine the percentage of NGFR expression.

Having optimised culturing conditions using lentivirus produced using the ‘standard’ protocol, we next determined the effect of combining the ‘final’ lentivirus generation protocol with the modified culture conditions. When these combinations were made and primary T cells were transduced with three independent lentivirus preparations we found that we could achieve high transduction efficiency, as measured by ΔNGFR expression (combined ΔNGFR expression 79.81 ± 14.68%) (Figure 5A), demonstrating the importance of fully optimised lentiviral production and transduction techniques.

## Conclusion

Lentiviral transduction of exogenous genes into primary cells is technically challenging, requiring the preparation of high titre viral stocks and optimal culture conditions. The generation of lentivirus requires the transfection of a stable producer cell line, such as 293T cells. We have shown that transfection of 293T cells with a second-generation lentivirus system produces virus with sufficiently high titre to transduce primary T cells, compared to the third-generation system. While this approach increased viral titre, the viral supernatants did not generate sufficiently high transduction efficiency. To increase the virus transduction efficiency we sought to concentrate the virus supernatant, thereby allowing for the use of a high MOI whilst at the same time reducing contaminating material in the supernatant. We determined that it was possible to increase virus transduction efficiency by concentration using ultracentrifugation. Transduction efficacy could be improved further by reducing the centrifugal speed from the standard protocol (90,000 g) to 20,000 g. In contrast, Acrodisc and Optiprep concentration and purification techniques gave little improvement over ultracentrifugation, and actually led to reduced viral titres and transduction of primary CD45RA^+^ T cells, potentially due to an increase in damaged virion particles. In addition to virus generation we also showed that transduction efficiency can be improved by modifications to cell culture conditions, specifically we showed that the strength of TCR stimulation and the time of transduction following stimulation affects transduction efficiency. Interestingly, we also showed that lentivirus can transduce unstimulated T cells to similar efficiency to stimulated primary T cells; however the expression of the transgene is low. Overall, we demonstrate an easy and robust protocol to generate and transduce lentivirus at high efficiency in primary T cells. This robust method is simple to achieve and does not require additional expensive laboratory equipment.

## Competing interests

The authors declare that they have no competing interests.

## Authors’ contributions

APC and AK performed the experiments and drafted the manuscript. APC performed the primary T cell transductions and analysed the data. AK generated lentivirus. BG participated in the design of the study and helped draft the manuscript. FMB conceived of the study, and participated in its design. All authors apart from FMB read and approved the manuscript.

## Supplementary Material

Additional file 1: Figure S1 Ultracentrifugation at 20 000 g improves the overall virus recovery. Virus recovery was measured before and after centifugation of lentivirus at decreasing centrifugal speeds. Virus recovery was then expressed as a percent of the total TU of starting unconcentrated lentivirus. *P > 0.05, **P > 0.01 as determined by Students t-test. Data is represented as mean ± s.d. of three independent experiments. **Figure S2.** Virus recovery is reduced following purification using Optiprep and Acrodisc. Virus recovery was measured before and after ultracentrifugation, Optiprep and Acrodic methods. Virus recovery was then expressed as a percent of the total TU of starting unconcentrated lentivirus. *P > 0.05, **P > 0.01 as determined by Students t-test. Data is represented as mean ± s.d. of three independent experiments. Click here for file
