# Supplementary material for: Simplified production and concentration of lentiviral vectors to achieve high transduction in primary human T cells
Source: BMC Biotechnol. 2013 Nov 12;13:98. doi: 10.1186/1472-6750-13-98 (PMC3830501; doi:10.1186/1472-6750-13-98)
Supplement: Additional file 1: Figure S1 — Ultracentrifugation at 20 000 g improves the overall virus recovery. Virus recovery was measured before and after centifugation of lentivirus at decreasing centrifugal speeds. Virus recovery was then expressed as a percent of the total TU of starting unconcentrated lentivirus. *P > 0.05, **P > 0.01 as determined by Students t-test. Data is represented as mean ± s.d. of three independent experiments. Figure S2. Virus recovery is reduced following purification using Optiprep and Acrodisc. Virus recovery was measured before and after ultracentrifugation, Optiprep and Acrodic methods. Virus recovery was then expressed as a percent of the total TU of starting unconcentrated lentivirus. *P > 0.05, **P > 0.01 as determined by Students t-test. Data is represented as mean ± s.d. of three independent experiments. [file 1472-6750-13-98-S1.docx]

**Additional file 1**

**Simplified production and concentration of lentiviral vectors to achieve high transduction in primary human T cells**

Adam P. Cribbs^1^, Alan Kennedy^1^, Bernard Gregory, Fionula M. Brennan^2^

^1^These authors contributed equally to this paper

^2^Deceased June 2012

Correspondence: [adam.cribbs@kennedy.ox.ac.uk](mailto:adam.cribbs@kennedy.ox.ac.uk)

Kennedy Institute of Rheumatology

Roosevelt Drive

Headington

Oxford

OX3 7FY

**Figure S1: Ultracentrifugation at 20 000g improves the overall virus recovery**. Virus recovery was measured before and after centifugation of lentivirus at decreasing centrifugal speeds. Virus recovery was then expressed as a percent of the total TU of starting unconcentrated lentivirus. * P>0.05, ** P>0.01 as determined by Students t-test. Data is represented as mean ± s.d. of three independent experiments

**Figure S2: Virus recovery is reduced following purification using Optiprep and Acrodisc.** Virus recovery was measured before and after ultracentrifugation, Optiprep and Acrodic methods. Virus recovery was then expressed as a percent of the total TU of starting unconcentrated lentivirus. * P>0.05, ** P>0.01 as determined by Students t-test. Data is represented as mean ± s.d. of three independent experiments
